# A novel and well-defined benchmarking method for second generation read mapping

**DOI:** 10.1186/1471-2105-12-210

**Published:** 2011-05-26

**Authors:** Manuel Holtgrewe, Anne-Katrin Emde, David Weese, Knut Reinert

**Affiliations:** 1 Department of Computer Science, Free University of Berlin, Takustr. 9, 14195 Berlin, Germany; 2 Max-Planck-Institute for Molecular Genetics, Ihnestr. 63-73, 14195 Berlin, Germany

## Abstract

**Background:**

Second generation sequencing technologies yield DNA sequence data at ultra high-throughput. Common to most biological applications is a mapping of the reads to an almost identical or highly similar reference genome. The assessment of the quality of read mapping results is not straightforward and has not been formalized so far. Hence, it has not been easy to compare different read mapping approaches in a unified way and to determine which program is the best for what task.

**Results:**

We present a new benchmark method, called Rabema (Read Alignment BEnchMArk), for read mappers. It consists of a strict definition of the read mapping problem and of tools to evaluate the result of arbitrary read mappers supporting the SAM output format.

**Conclusions:**

We show the usefulness of the benchmark program by performing a comparison of popular read mappers. The tools supporting the benchmark are licensed under the GPL and available from http://www.seqan.de/projects/rabema.html.

## 1 Background

Second generation (2G) sequencing technologies have many and diverse biological applications [1-7] and have effectively transformed the field of DNA sequence analysis. With the advances in sequencing technologies that continuously increase throughput at dramatically decreasing costs, also an increased demand for computationally efficient analysis tools has arisen. One of the most demanding but fundamental computational processing steps is read mapping, i.e. finding the positions of all sequenced reads in a reference genome. A variety of tools to solve the read mapping problem have been published, e.g. [8-12]. As read mapping is fundamental to all downstream analyses, the outcome of an analysis may differ significantly depending on the way reads were mapped. In addition, research in this field will remain active due to continuous progress in sequencing technology. Hence, the need for a careful and clear definition of the quality (resp. accuracy) of a read mapping result is apparent. Also, as the number of users of 2G sequencing machines and the number of read mapping tools for different purposes increase, it becomes crucial to be able to compare read mapping software to determine what tool is the best one for a specific purpose. Unfortunately, the various read mappers have different properties and use slightly different definitions of the read mapping problem which make such a comparison difficult.

Here, we will discuss and carefully define this problem, and point to the challenges that comprehensive and sensitive read mapping faces. Furthermore, we present a novel benchmark based on which the quality and speed of read mapping tools can be assessed. Our contribution consists of a *precise definition *of the read mapping problem and *tools *to evaluate the results of a read mapper. This enables the generation of gold standards for both simulated and real-world reads. It thus overcomes shortcomings when only using simulated reads, such as biases present in real data (cf. [13]). Using the four read mappers shown in Table S1 (supplementary tables, figures, and sections can be found in Additional File 1), we give an example of such an evaluation.

Besides helping to objectively compare programs, proper benchmarking has other merits, namely to kindle a keen competition among computer scientists. This often results in efficient algorithms and fast implementations. Examples are the RNASeq Genome Annotation Assessment Project [14] or the ENCODE Gene Prediction Workshop [15] which resulted in many new approaches to gene prediction and quantification.

Another advantage is better support for the algorithm engineering cycle [16]: First, an algorithm is designed and analyzed theoretically. This is followed by a careful implementation. The implementation is then experimentally evaluated and the theoretical hypotheses verified or discarded. Using this information, the next iteration in the cycle can be started.

Careful experimentation is one key aspect of a successful practical solution to algorithmic challenges. We expect that our benchmark method will help to advance read mapping research in the next years.

## 2 Methods

We use common notation from mathematics and computer science. We denote closed ends of an interval with square brackets and open ends of an interval with round brackets. For example, [*a*, *b*) is a half-open interval with the values from, and including, *a *up to, and excluding, *b*.

For a sequence *S*, *S^rc^* gives the reverse-complement by reversing *S *and exchanging the characters with their complement. As usual, in DNA: C is exchanged with G and A with T.

### 2.1 The Read Mapping Problem

An abstract definition of the read mapping problem can be given as follows. The input is a reference sequence *S*, a set *R *of reads *r*, a distance function *δ *and a maximal distance *k*. *δ *assigns a distance to semiglobal alignments of reads against *S*. The domain of *δ *determines which alignments are possible, e.g. whether Hamming or edit distance is used. Note that *δ *could also be the score of an alignment (e.g. a Smith-Waterman score), which we do not consider in this paper (see Section S3).

For each read *r*, the problem is to find a set of *matches *of *r *in *S*. The precise definition of the term *match *is suprisingly involved and will be given in Sections 2.2 to 2.4. For now, let a match be *a location in the reference where the read aligns*. A *feasible *match is a match where the read aligns with distance ≤ *k*. A *best match *is a feasible match that has the smallest distance of all feasible matches for the given read. We can rank the matches ascendingly by their distance. Now, let us consider the set of matches that is to be found. Obvious choices for the match set could be to find: (1) all feasible matches, (2) all best matches, (3) up to *c *best matches, or (4) up to *c *best-ranking matches. In this work, we consider (1-3), and (3) with *c *= 1, refered to as *all, all-best*, and *any-best*.

From the application of read mapping in biology, the *biological problem *arises. Here, the position in the reference should be found that corresponds to the sample position of each read. Because of ambiguities, this problem cannot be solved directly, but is approximated by the mathematical problem.

### 2.2 Defining Matches

In this section, we will try to give an intuition for the difficulties inherent in defining matches. These difficulties stem mostly from the problem of how to decide when two matches are *different *and when they should be considered the *same*. This will profoundly influence how we define a *match *and thus how we count correct matches.

First of all, we do not allow the first and last base of a read to align with a gap in the reference sequence. Such alignments are superfluous: Aligning the first/last base to the base at the left/right of the gap will always yield an alignment with a lower or equal distance. Figure S4 gives an example.

#### When Are Two Matches Different?

When publishing their work, many authors of read mapping software simply count the number of mapped reads. This only allows for a crude assessment of read mappers relative to each other but not to the best possible solution.

**Figure 1:**
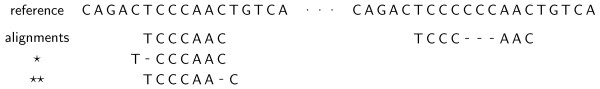
**Alignments of the read TCCCAAC against two locations in the reference sequence**.

**Figure 2:**
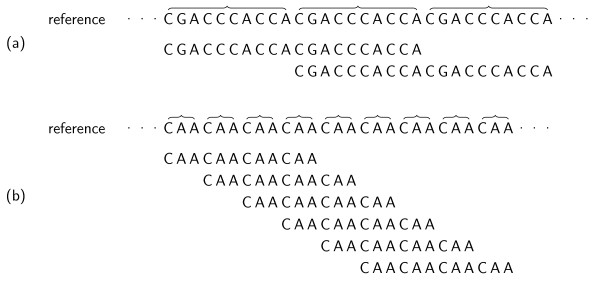
**Two examples for reads mapping in a long (a) and in a short (b) tandem repeat**.

Additionally, special care has to be taken when considering uniquely matching reads: If the read mapper does not have full sensitivity, it could miss a second match of a read and report it as unique match. Another read mapper could find both matches and discard the read as non-uniquely matching. In this case, a less sensitive read mapper could get a higher rating. Thus, one would also have to compare the non-uniquely matching reads as found by a read mapper to the ones reported as uniquely matching and compute a set of reads that are false positives. This is rarely, if ever, done in the literature, though. Note that this set of false positives can only be seen as an approximation if no read mapper with full sensitivity is included in the comparison. Additionally, a definition of "full sensitivity," i.e. of a gold standard is still required. Consider the read and reference sequence fragments from Figure 1. Say, we want to find the best two matches of the read in the reference sequence, with an edit distance of up to 3. Both with an edit distance of up to 3. Both localocations in the reference sequence are shown. The row *alignments *shows two alignments of the read to the reference sequence that appear to be "natural." However, the alignments in rows ⋆ and ⋆⋆ have a lower edit distance than the right one. Common sense would tell us that the alignments in the left column are not "significantly different," though. Each alignment with distance *k *induces alignments with distance at most *k *+ 2 by aligning the leftmost/rightmost base one more position to the left/right and introducing a gap. Repeats are another issue. Consider the tandem repeat in Figure 2(a). Intuitively, we can identify the two distinct alignments shown there. Figure 2(b) shows another tandem repeat of shorter period with a read that aligns in this repeat region. Searching for alignments in Figure 2(b) in the same way as in Figure 2(a)), we could identify all alignments given in this figure.

However, counting alignments in this way would require a read mapper to find lots of positions in repeat regions. This is not desirable since reads from long tandem repeat regions would get a higher weight with this counting scheme than reads from short tandem repeat regions or reads from non-repeat regions. Only weighting each found match with ^1^/*_n_* (where n is the number of positions the read aligns at) is deficient, too. It would be preferable to find a way to naturally *merge similar matches *(e.g. the one from the left column of Figure 1), matches that are very close to each other (c.f. Figure 2(b)) and to *separate matches that are sufficiently distinct *(c.f. Figure 2(a)).

To give a clear description on how to separate matches, we will first introduce *trace trees*.

#### Trace Trees

Consider a dynamic programming matrix for semi-global alignment (cf. [17]). Each alignment is represented by a path from the top row to the bottom row. Horizontal and vertical movements between cells represent indels, diagonal movements matches and mismatches.

Standard DP alignment algorithms yield the smallest distance for each alignment end position. From an end position, we can search for start positions by performing a traceback search backwards/upwards in the matrix. Given a value for *k*, we can find all start positions for the given end position that yield alignments with distance ≤ *k*. The backward search yields a path through the matrix which we call *trace*.

**Figure 3:**
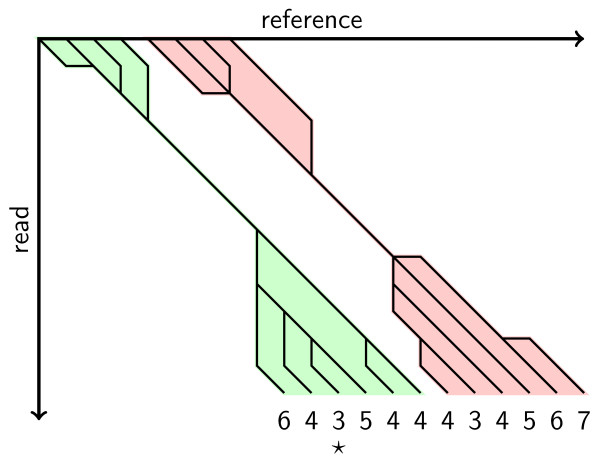
**This figure shows two neighbouring trace trees**.

Note that we only consider DP algorithms that are deterministic and always perform the same choice in case of ambiguities. For example, if they have the choice between tracing back vertically, diagonally, and/or horizontally, they could always take the rightmost choice. In this case, they would prefer vertical over diagonal, diagonal over horizontal movement. Needleman-Wunsch is one example of such an algorithm. When plotting the traces for all feasible matches, we could get an image like the one shown in Figure 3 (The numbers below the lower leafs in Figure 3 give the minimal distance for the best alignments ending in this position): We can consider the traces as graphs where cells correspond to nodes and movements in a trace between cells can be seen as edges. The resulting graphs have some simple properties, namely that a) the graph decomposes into connected components and b) each connected component is a tree. If one chooses any vertex on the trace shared by all alignments as a root, then the resulting rooted tree is split into an upper and a lower part. The upper leafs correspond to possible start positions of alignments, the lower leafs correspond to possible end positions. Each combination of one upper and one lower leaf corresponds to a specific (although not necessarily feasible) alignment and is thus an upper bound on the number of feasible alignments.

#### Hamming Distance Matches

If we want to count all possible alignments, we note that each match in the Hamming distance model corresponds to exactly one diagonal in the matrix, namely the one between the start and end position of the match. Thus, we can define a match with Hamming distance simply with either its start or end position. For consistency with our choice for edit distance (see below), we pick the end position.

#### Redundant Edit Distance Matches

Considering all combinations of start and end positions is not desirable for edit distance: In Figure 3, there would be 4 × 6 = 24 such matches in the left tree alone, possibly many feasible ones. We have to resort to other means for counting alignments in the case of edit distance.

#### Identify Matches With End Position

We observe that the shared trace is usually longer than the branching parts. This means that large parts of the alignment are basically the same and even differing alignments might have the same distance. To avoid counting these as separate alignments, we proceed as follows.

We identify each match with its end position *e *and use the leftmost start position *s *with minimal distance as its *canonical *start position. The choice of *s *as the canonical start position is arbitrary. However, picking the leftmost position as *s *has the advantage that the interval between *s *and *e *contains the start position of all alignments of minimal score ending in *e*. In the example from Figure 3, this reduces the number of matches for the right tree from 24 to 6.

### 2.3 Error Landscapes

**Figure 4:**
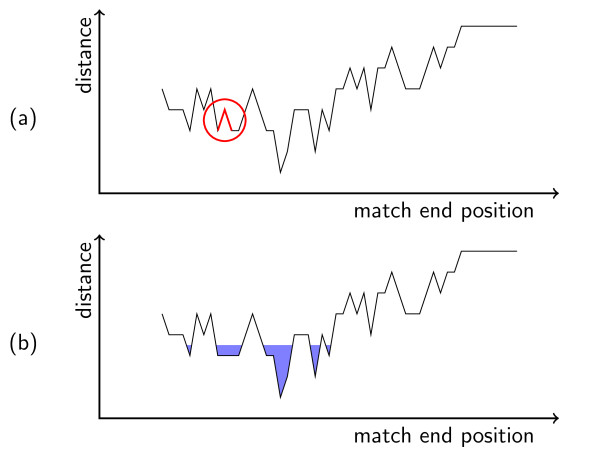
**This figure gives an example of the error landscape: (a) shows the landscape before smoothing and (b) shows it after smoothing and with water**. The end position is plotted on the x axis, the distance is plotted on the y axis. In (a), the raised ground water is shown and the separating position has been smoothed. The point between the lines marked in red is a separating position (see Definition 3).

In this section, we define *error landscapes *in order to capture the intuition of the match definition we will give in Section 2.4 more formally. The distance *δ*(*i*) of a read to the genome at position *i *is the distance of the best alignment that ends there. If we plot the points (*i*, *δ *(*i*)) for each reference sequence position *i *and connect them, we get an error landscape such as the one shown in Figure 4(a). In this landscape, valleys represent regions where the read aligns with a low distance and mountains represent regions where the read aligns with a high distance.

Now, we let imaginary ground water in our landscape rise to a level of *k *+ 0.5. This is shown in Figure 4(b). In this example, this yields five lakes. Each lake represents a class of matches with sufficiently low distance. The metaphor of the landscape with lakes corresponds to the natural merging of similar matches.

We expect a read mapper to locate each of these classes but it suffices to find one representantive in each class for the *all *variant. For criteria *all-best *and *any-best*, each lake is assigned the distance of the point with the lowest distance of all contained points. Put differently, each lake is assigned its depth – if we stay in the metaphor of landscapes and lakes.

### 2.4 Matches as Equivalence Classes

After arguing, which matches should be considered the same and which different, we need to formalize this notion. Hence, the aim of this section is to give a strict mathematical definition for the term *match *such that it closely models the intuitions from Sections 2.2 and 2.3.

In Section 2.2, we already argued that we want to identify each match with its end position. We also enforce the alignment of the last read and reference bases as described there. Now, we want to find an equivalence relation that partitions the set of feasible matches in a sensible way such that each class corresponds to an intuitive match.

We will do this by defining an equivalence relation for merging neighbouring matches and then defining another one that merges separated feasible matches sharing the same trace. For numbers *a*, *b *in the following, we assume w.l.o.g. that *a *≤ *b*. Also, we identify matches with their end position and use the two terms match and end position interchangeably.

**Definition 1 **(Neighbour Equivalence). *Two feasible matches (identified by their end positions) a, b are *neighbour equivalent * if for all x, a *≤ *x *≤ *b the following holds: δ*(*x*) ≤ *k*.

**Definition 2 **(Trace Equivalence). *Two matches a*, *b are *trace equivalent *if their traces share a part. This is the case if their canonical start position is equal*.

For example, for *k *= 4, the last match ending in the rightmost leaf of the left tree and the leftmost leaf of the right tree in Figure 3 are neighbour equivalent but not trace equivalent. However, the matches ending at the third and fourth leaf of Figure 3 are trace equivalent but not neighbour equivalent.

**Definition 3 **(*k*-Trace Equivalence). *Two matches a, b are k*-trace equivalent *if one of the following holds: (1) They are feasible, neighbour equivalent, and trace equivalent. (2) There exist feasible, trace-equivalent matches α, β and a separating match ζ such that α *≤ *a *≤ *ζ *≤ *b *≤ *β*.

*A *separating match *ζ is a match with δ *(*ζ*) *> k and there exists α, β, α *<*ζ *<*β such that δ *(*α*), *δ *(*β*) ≤ *k*.

Obviously,  and  are equivalence relations. Also, it is easy to see that  is reflexive, symmetric and transitive and thus an equivalence relation. We now define two matches *a*, *b *to be equivalent (*a *≡ *b*) if they are *k*-trace equivalent or neighbour equivalent. The disjunction of two equivalence relations yields another equivalence relation.

It follows that ≡ gives a well-defined partition of the feasible matches which corresponds to the intuitions given in Section 2.3.

### 2.5 Gold Standard and Evaluation

Following the definition of *k*-trace equivalence, each equivalence class is an interval. The reference data set (*gold standard*) can thus be described as an array of triples (*k*, *first*, *last*) describing all intervals of feasible matches [*first*, *last*] for a given *k *for each read.

Given the gold standard and the result of a read mapper, the quantitative evaluation of the read mapper result is easy. In the evaluation, a specific value is selected for *k*, say *c*. Now, all intervals from the gold standard are selected, where the value of *k *equals *c*. After sorting these intervals, binary search can be used to check which equivalence classes were found by the read mapper.

An additional preprocessing step has to be done in the case of *all-best *and *any-best *evaluations. Here, we update the value of *k *in each interval *I *in the gold standard to the smallest value of *k *for all intervals contained in *I*. This is done before the selection step described in the above paragraph.

Further technical issues are described in Section S1.

### 2.6 Building the Gold Standard

We differentiate between building the gold standard for the biological problem and the mathematical problem.

#### Biological Problem

Since it is not possible to observe the sequencing process at the molecular level, we use simulated reads. Note that simulation data always has certain shortcomings, as biases present in real biological data are hard to simulate. Such biases in short read sequencing data have been reported e.g. in [13]. Nevertheless, simulated data can be informative in benchmarking tools, and can therefore be used to complement real-world data.

From our simulation, we obtain read sequences together with their actual sample positions. Each of these positions is a representative of the one equivalence class the read mapper should find. Given this representative, the whole equivalence class (i.e. interval) can be found as described below for the mathematical problem. This procedure is, in essence, similar to simulating reads and checking whether their mapping position is close to the actual sample position, but has the advantage of not having to choose a cutoff for what is defined as "close". With our definition, the genomic sequence itself defines how far away from the originally simulated position a read may map in order to be counted as correct. Intervals in ambiguously mappable regions will be broader, while intervals in unambiguous regions will be tight.

#### Mathematical Problem

A naïve solution for generating a gold standard for the mathematical problem is to use an online multiple string search algorithm and then merge the matches, according to ≡. However, this is too slow even for moderate genome sizes.

A more sophisticated method is to take the matches of a read mapper with full sensitivity as the input. This will yield at least one match *m *in each equivalence class. Using *m *as seed, we can then reconstruct the interval around it and only have to look at a fraction of the reference sequence.

Starting from each *m*, we first extend the interval to the right. We extend until we find a match that has score >*k *and no match right of it with score ≤ *k *that has the same begin position. Analogously, we extend the interval to the left.

Finding the end and begin positions of the alignments can be efficiently implemented with approximate string search algorithms for Hamming and edit distance. For edit distance, we use Myers' bit vector algorithm [18], for Hamming distance we use a naïve implementation.

Given *k_max_*, a maximal value for *k*, we compute the gold standard for all 0 ≤ *k *≤ *k_max_* for each read.

### 2.7 Read Mapping and Similar Problems

The mathematical objective of read mapping may vary for different types of biological analyses. For example, when mapping RNA-Seq reads onto a genomic sequence, one should consider that reads will span exon-exon boundaries. Here, a spliced mapping approach would be a reasonable choice.

The benchmarking method that we describe here considers the "core" read mapping problem, and evaluates how far a read mapper is away from the mathematically optimal solution. We do not address related problems such as spliced read mapping or multi-read assignment. We only consider pairwise alignments for individual reads using the popular and parameter free distance measures Hamming and Levenshtein distance.

Still, being able to measure how sensitively a read mapper detects all (best) mapping positions is indirectly useful for multi-read assignment: If a read mapper misses a high number of mapping locations, a subsequent multi-read assignment step is less likely to find the correct assignment.

### 2.8 Practical Considerations

The description above is simplified in some parts to ease the understanding. In practice, there are the following differences and additional considerations:

We always used absolute error values in our descriptions which is appropriate for reads of the same length. However, some technologies, e.g. 454 pyrosequencing, yield reads of varying length. Thus, we use error rates, which are relative to the read length.

Gold standards could be built from any read mapper with full sensitivity, e.g. Mrsfast [19] or Razers [12]. Razers supports both Hamming and edit distance for arbitrary read length whereas Mrsfast only supports Hamming distance. Of course, also tools that claim 100% sensitivity may contain bugs; RazerS is our in-house tool that we can correct quickly in case of problems. Therefore, we chose Razers for building gold standards.

### 2.9 Read Simulation

For our benchmark, we use the Mason read simulator [20]. The program takes a FASTA genome reference sequence *S *for its input. It then simulates an arbitrary number of haplotypes by adding indels and mismatches to *S*. Third, it simulates read sampling from the haplotypes, depending on the sequencing technology. Finally, it writes out the reads in FASTQ files and also creates a SAM file that describes the gold standard for the biological problem from Section 2.1.

Details can be found in Section S2.

## 3 Results and Discussion

### 3.1 Read Mapper Comparison

We have evaluated the read mappers Bowtie [11], Bwa [9], Shrimp2 [10], and Soap2 [8] on read sets for D. melanogaster and S. cerivisae from the Short Read Archive (SRA). Information about the read sets can be found in Table S2, Table S3 shows information regarding the used reference sequences. The gold standard for the mathematical problem from Section 2.1 was built with an error rate of 8% and edit distance. Also, we generated simulated read datasets for the evaluation of the biological problem from Section 2.1.

We used default parameters for Bwa as advised by the author; Illumina reads were mapped using the commands *aln, samse*, and *sampe*, 454 reads were mapped using *bwasw*. For Shrimp2, weighted seeds were used to improve performance for longer reads, as suggested by the authors. For Soap2 and Bowtie, we performed some initial benchmarks to optimize sensitivity, at the cost of a higher running time. These programs were also run with default paramters, the variants with tuned parameters are labeled Soap2 and Bowtie*. For parametrization details, see Section S4.

We limited the output of each read mapper to 100 alignments per read, where possible, to 1 for simulated reads. There is no option to limit the output of Soap2 to a certain number of alignments per read. For the evaluation, we perform a postprocessing step and only select the best 100 matches by edit distance, breaking ties randomly.

The experiments were performed on a computer with Linux 2.6.30, Intel Xeon processors with 2.67 Ghz and 76 GB of main memory. No program was run with more than one process/thread. Memory consumption was measured by parsing the output of the Unix command top every second. Table S4 shows the resource consumption for building the indices of Bowtie, Bwa and Soap2.

As a sanity check of the method, we also ran Razers with default parameters on all read sets. The expectation was that it should find nearly all intervals since it uses the same definition of the read mapping problem as the read mapping benchmark. Full sensitivity should only be limited by (1) reads with more than 100 matches, (2) the default sensitivity of 99%, and (3) its default error rate of 8% which might make it join lakes that are separated when analyzing with a lower error rate. The running time was expected to be generally lower than that of Shrimp and higher than that of index-based tools. This expectation was fulfilled and subsequently, Razers was excluded in the following evaluation. Plots and data that also show the performance, as well as the running times of Razers are available from the project homepage.

The metric *normalized found intervals *is defined as follows: Each read gives at most one point. If a read matches at *n *locations (i.e. intervals), each found location gives ^1^/*n *point. To get percentages, the number of achieved points is divided by the number of reads and multiplied by 100. In the following, we will use the terms *sensitivity *and normalized found intervals interchangeably.

#### Real-World Data

**Figure 5:**
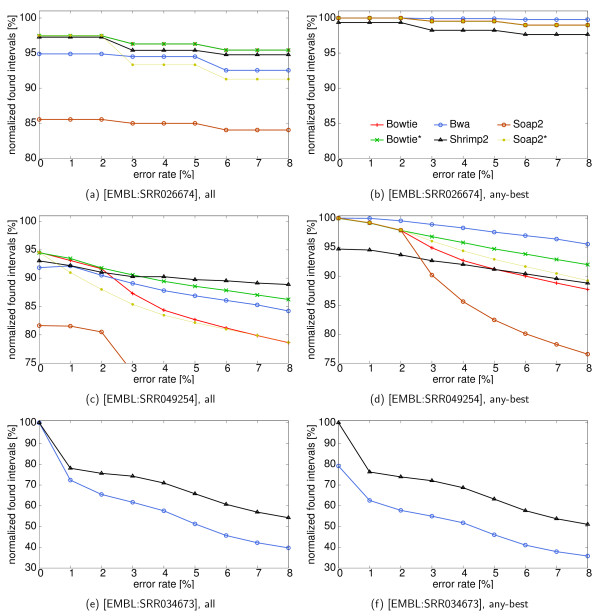
**Normalized found intervals (percentage, categories *all*/*any-best*) for different programs on reads from fly**. The gold standard was generated with an error rate of 8% and edit distance. The programs were run with default parameters, the x axis shows the variation of error rate in the evaluation. Read set properties are as follows. [EMBL: SRR034673]: 454 reads, average length 273 bp; [EMBL: SRR026674]: Illumina reads, 36 bp; [EMBL: SRR049254]: Illumina reads, 100 bp. The key is the same for all plots. Note the different axis scalings.

Figure 5 shows the evaluation of normalized found intervals (aiming to find *all *and *any-best *intervals) over a varying error rate using different read sets for D. melanogaster. The plots are for 10,000 uniformly sampled reads from each read set. The read sets' SRA accession numbers are given in the caption of each plot. Table S5 shows the running time and memory consumption of the programs on these read sets. Here, we want to focus on the evaluation of sensitivity. Bowtie was not run on 454 reads because its lack of support for gaps practically makes it inapplicable to the indel-error prone 454 reads. Likewise, we did not process 454 reads with Soap2 since we were not able to obtain suitable parameters.

Figures 5(a) and 5(c) show the sensitivity results for the *all *problem on Illumina reads. Bowtie* and Shrimp2 are the most sensitive tools; while all tools' performances suffer from increasing error rates, Shrimp2 achieves the highest sensitivities at high error rates where the absolute number of errors is greater than 3. Soap2 and Bowtie, especially in their default versions, seem to be tuned toward low numbers of errors. The effect can be seen on long reads in Figure 5(c): Using default settings, both tools' sensitivities drop dramatically for reads with more than 2 errors. The optimization of parameter settings for Soap2 and Bowtie clearly improves performance on long Illumina reads. For high error rates, this improvement even leads to 10 percentage points more sensitivity. For the short reads in Figure 5(a), Bowtie's default already constitutes the optimal parameter setting; the lines for Bowtie* and Bowtie are therefore the same here. From Figures 5(a) and 5(c), we also see that Bwa does not perform very well in the *all *category. It is consistently about 2-3 percentage points behind Bowtie*. This is explained by the fact that Bwa only reports a single match for reads that exceed the number of matches to report (in our case 100).

Looking at the results for Illumina reads in the *any-best *category shown in Figures 5(b) and 5(d), we see that Bwa is the best performing tool for this case. This holds for long and short reads, and for all investigated error rates. For short reads, both versions of Soap2 and of Bowtie perform equally well. As they are fully sensitive for reporting at least one best Hamming match for each read, their sensitivity only drops here due to missed gapped alignments. As can be seen for long reads, an absolute number of errors greater than 2, again leads to an increase in missed matches for the default versions of Soap2 and Bowtie. While Shrimp2 performs very well at the *all *problem, in the *any-best *criterium it lags behind all other tools (with exception of the non-optimized default of Soap2 and also Bowtie's default at high error rate). Due to the limitations mentioned above, only Bwa and Shrimp are shown in Figures 5(e) and 5(f). For the long 454 reads, there usually exists only one or few mapping locations per read. Therefore, differences in sensitivities between the *all *and the *any-best *category are not as pronounced as for the shorter, more ambiguously mapping Illumina reads. Here, Shrimp2 consistently has a lead of 10-20 percentage points over Bwa. This higher sensitivity comes at the cost of one order of magnitude higher running time and memory consumption.

We conclude from our analysis that Shrimp2 is a highly sensitive tool for detecing multiple matches (category *all*). Thus, it appears to be a good choice for analyses that require high sensitivity. Also, Bwa is a very diverse tool and shows especially good performance for the practically relevant *any-best *problem. Apparently, Bowtie and Soap are geared towards fast short read mapping with low error rates. In the *any-best *category they find matches of short reads with high sensitivity.

For all tools, parametrization becomes increasingly important with increasing read lengths and increasing numbers of errors. Different parameter settings for the same tool can lead to discrepancies in sensitivity of more than 20 percentage points. This emphasizes the importance of a benchmark such as the one presented in this article which can be used by developers and users alike to test tools with different parametrizations. Figures S6 and S7 show the same evaluation for the data on reads from S. cerivisiae. (Note that tests were only performed on Illumina reads of length 20 and 36 since no longer reads could be obtained for this organism.) The relative results and conclusion are similar for all read sets; sensitivies are higher for all read mappers, due to the lower repeat content of the genome. Notably, Shrimp2 does not gain as much as the other read mappers on the Illumina reads, but still achieves a high sensitivity.

#### Simulated Data

Tables 1 and S7 show the sensitivity of the read mappers on simulated data for fly (and yeast). Bwa and Shrimp2 consistently yield the best results, finding the best locations of at least 90% of all intervals on all read sets. The results for yeast reads are better than for fly reads. The most likely explanation is the lower complexity of the yeast genome with less ambiguities. Both read mappers' quality *increases*, with increasing read length, probably because of the same reason: The longer the reads are, the less ambiguities there are.

**Table 1:** Performance in found interval percentage for the read mappers on simulated D. melanogaster reads.

	Illumina 36 bp	Illumina 50 bp	Illumina 100 bp	454 Ø 200 bp	454 Ø400 bp
Bowtie	78.5	72.2	55.0	-	-
Bowtie*	78.5	72.2	59.2	-	-
Bwa	92.7	93.5	93.8	-	-
Shrimp2	90.1	91.1	92.8	89.7	92.9
Soap	79.6	73.1	54.7	-	-
Soap*	79.8	73.1	57.5	-	-

Data is given for mapping in edit distance mode. 454 reads were only mapped with Shrimp2.

Bowtie and Soap2 do not support indels and are consequently not robust against the rising number of indels in the longer reads. This can be seen in the decreasing quality of their results. The optimized parametrizations yield slightly better results than the default parametrizations. In total, Bowtie finds slightly more original locations than Soap2, probably because of support for base qualities.

### 3.2 Usages For Our Method

Our method is very useful for the *exact *validation of read mapper results. It can be used to compute the exact percentage of matches found by a read mapper. This can be done for large samples of read data sets, 10,000 in our example, but more are possible.

For performing a comparison of read mappers, we propose the following guidelines:

1) Use reads from state-of-the-art technology with popular parameters for size and paired-end modes. 2) Use current versions of popular tools from multiple paradigms, such as index-based filtration-based read mappers. 3) Run the read mappers with various parametrizations, including default parameters, possibly allowing the read mapper authors to provide the best possible parameters. 4) Use a method based on a formal definition, e.g. Rabema, to perform an exact assessment of read mapper quality. 5) Complement this with heuristic measures such as counting mapped and uniquely mapped reads for datasets of real-world-size, taking into considerations the notes in Section 2.2 about possibly missed duplicate matches. 6) Possibly, show how the results of downstream analysis differ between two read mappers. 

Our method gives a *gold standard *for the read mapping problem. This works for both simulated and real-world read sets and allows to put each read mapper not only in relation to other read mappers, but to an optimal solution.

Furthermore, the implementation of our method allows to print missed equivalence classes/intervals. This allows the analysis of why a read mapper does not find certain matches. It can also be used to debug and improve read mappers as well as evaluate the automatic computation of read mapper parametrization. If a read mapper finds a location that is not in the generated gold standard then this is reported by our tool as well and can be seen and used as a sanity check.

## 4 Conclusions

We presented a benchmark for read mapping, beginning with the distinction of the biological problem and a mathematical abstraction. For the mathematical abstraction, we gave a precise problem definition which allows to define the required results. Our method works both for real-world reads and simulated data.

We implemented tools to build the introduced gold standard and performed a comparison of several popular read mapping tools. The example comparison uses Illumina and 454 reads, both real-world and simulated data. We found that Shrimp2 is a highly sensitive tool for detecting multiple matches. Bwa is a very diverse tool and especially good for finding one of the best alignments of a read. Soap2 and Bowtie are both good choices for mapping short reads quickly and sensitively, Bowtie being a slightly better choice according to our analysis.

Currently, our method is limited to base-space reads. However, three of the four currently commercially available 2G sequencing platform (including the widely used Illumina technology), create reads in base space. Thus, our method is useful for a wide audience.

The online material at http://www.seqan.de/projects/rabema.html contains download links for the reference sequences and read sets we used, the resulting SAM files, the tools for the benchmark evaluation, and a manual.

### 4.1 Future Work

At the moment, the generator for the gold standard does not incorporate mate pair information and quality values. We plan to add support for this in a future release. Note that read mapper programs incorporating mate-pair and quality value information can already leverage this information in benchmarks for the biological problem.

Another point for improvement is allowing to use ABI SOLiD [21] reads. For this, support for color-space sequences has to be added to SeqAn, the gold standard generator has to be adapted to support them and Razers (or another exact read mapper) has to be extended to work with color-space reads. More details on this can be found in Section S3.

## 5 Authors' contributions

The original idea of the benchmark came from DW and KR. DW came up with the intuition of the error landscape. From this idea, MH derived the exact definition through equivalence classes, trace and neighbour equivalency, implemented the software and wrote most of the paper. AKE helped with the experimental evaluation. Additionally, AKE, DW and KR contributed equally to the work through discussion and editing.

All authors read and approved the final manuscript.

## Supplementary Material

Additional file 1 **Supplemental Material**. This file contains supplemental text, figures, and tables. Click here for file
