# Supplementary material for: A novel and well-defined benchmarking method for second generation read mapping
Source: BMC Bioinformatics. 2011 May 26;12:210. doi: 10.1186/1471-2105-12-210 (PMC3128034; doi:10.1186/1471-2105-12-210)
Supplement: Additional file 1 — Supplemental Material. This file contains supplemental text, figures, and tables. [file 1471-2105-12-210-S1.PDF]

Supplementary Material for "A Novel And Well-Defined Benchmarking Method For Second Generation Read Mapping"

Manuel Holtgrewe, Anne-Katrin Emde, David Weese, and Knut Reinert

March 2010

S1 Technicalities

In this section, we describe some more involved details of the benchmark.

S1.1 Ghost Matches And Landscape Smoothing

We note that each match with distance $\leq k - 2$ implies at least one match on both sides of it. Figure 1 in the main article shows an example. This can also be seen in Figure 2 in the main article. For $k = 5$, the third end position of the third lower branch in the left tree implies feasible matches left and right of it. This problem is partially solved by the definition of neighbour equivalence in Section 2.4 of the main article.

However, there is a problem when merging matches in this way: For $k = 4$, the end position marked with $\star$ (at the fourth lower branch of the left tree) separates the matches left and right of it. However, in Section 2.4 of the main article, we explained that alignments sharing their trace are basically the same. Thus, it is desirable to merge the matches left and right of such *separating positions*. This is the reasoning behind defining trace equivalence and combining it into $\equiv$.

S1.2 Matches On The Reverse Strand

We observe that there is a pecularity with this definition when searching for reverse-complemented reads or reads on the reverse strand of the reference sequence. An example for this is shown in Figure S2 for $k = 5$. Because of the asymmetry of the search (forcing the alignment to end at each position while chosing the best begin position), there are two lakes in the first case but only one lake in the second case. Note that there is a truly separating match in Figure S2a, the match with distance 6. No two matches in the lake left and right of it are in relation $\equiv$.

reference C C A G C G - A G A T distance = 2

X X

read G C G C

(a) Ghost match left of the match in Figure S1b

reference C C A G C G - A G A T distance = 0

X

read G C G C

(b) A perfect match of a read.

reference C C A G C G A G A T distance = 2

X X

read G C G - C

(c) Ghost match right of the match in Figure S1b

Figure S1: This figure shows a perfect match of a read in the reference sequence and two ghost matches left and right of it.

When building the gold standard, we can search for the reverse-complemented reads in the original reference sequence or search for the original reads in the reverse-complemented reference sequence. Depending on this choice, there might be more or less equivalence classes. In practice, we observed very few differences, mainly in low-complexity repeat regions and with very long (e.g. 454) reads only.

We build the gold standard using Razers. Razers reverse-complements the reference sequence because this is computationally more efficient for its approach. The situation from Figure S2a might occur at some place. A read mapper that reverse-complements the reads (index based read mappers such as Bowtie and bwa do so) will only see one lake, as depicted in Figure S2b.

However, since this almost only occurs in low-complexity regions and the number of occurences is small, the resulting difference of results will be neglegible. Furthermore, such read mappers can easily implement a special benchmark mode that reverse-complements the reference sequence instead of the reads. This might come at a higher computational cost but can then be used to assess the sensitivity and verify that the bias against the approach of reverse-complementing the contigs is small. For measuring computational costs, the normal mode can be used.

Note that one has to choose whether to reverse-complement the reads or the reference sequence. Each decision would slightly bias towards the chosen approach. We chose to reverse-complement the reads since this is what Razers does. When another read mapper with full sensitivity for Hamming and edit distance is released, an alternative decision could be made.

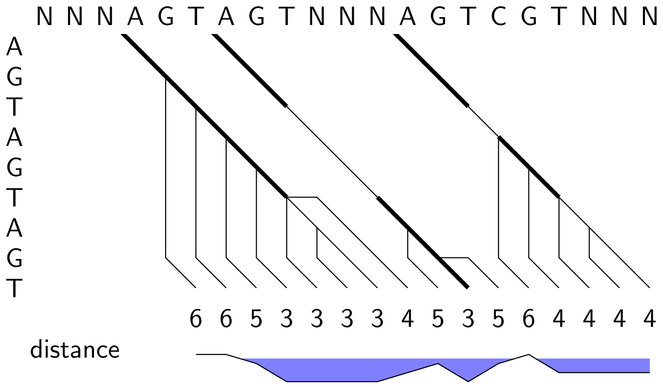

(a) Forward strand.

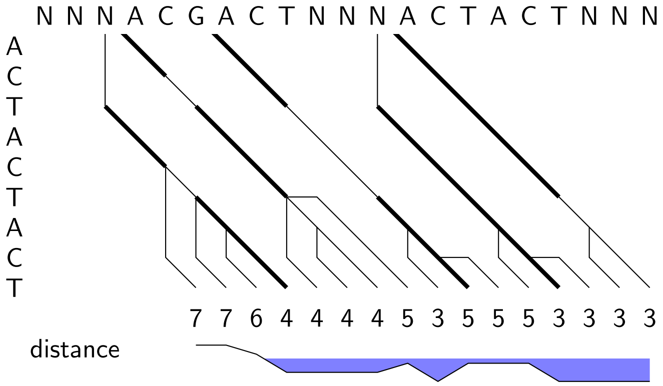

(b) Reverse strand

Figure S2: This figure shows a pecularity with matches on the reverse strand. Depending on whether the reference sequence or the reads are complemented, there might be an infeasible match that separates two match classes. Thick lines correspond to matches whereas thin lines correspond to mismatches or indels.

S2 Read Simulation Details

S2.1 Illumina Reads.

For Illumina reads, the simulator follows a simple statistical approach.

In a precomputation step, we aligned a set of reads against the reference genome with Razers using full sensitivity and allowing an error rate of up to 8 %. The best matches for each read are returned. The resulting SAM alignment file is then analyzed for position specific substition and indel rates as well as position and error dependent quality distributions. For more information on this procedure, see [1].

From this, we derive the following, simple error model. (We are not aware of any other read simulator that is able to simulate qualities position- and error-dependent.)

Insert and deletion rates are not position dependent and set to 0.1 %. Error rates are described by a piecewise linear function. It rises from 0.2 % at position 0 to 0.26 % at position 0.66×read length. From there, it rises to 1.2 % for the last position. The qualities are modeled by a normal distribution, one for substituted and one for non-substituted bases. The means and standard deviations are linear functions of the position. Means fall linearly from 40 to 39.5/from 39.4 to 30, standard deviations rise linearly from 0.05 to 10/from 3 to 15 from the first to the last position for substituted/non-substituted bases.

In the simulation step, an infix of the haplotype is sampled uniformly at random. Then, an edit string is simulated, depending on the error probabilities for each read position as computed in the precomputation step. Possibly, the infix size is updated in this step if the number of inserts and deletes in the edit string is not the same, such that the resulting simulated reads have the same length. This is followed by applying the edit string to the haplotype infix, yielding the simulated read. Finally, the quality value for each read position is generated, also based on the model described above.

S2.2 454 Pyrosequencing Reads.

For 454 reads, the simulator follows the approach also taken in [2,4]: It samples uniformly distributed infixes of normally distributed length from the haplotype. Then, it does a simple flow cell simulation. Light intensities are simulated with normal distributions, noise with lognormal distribution. The quality values are log-odds scaled error probabilities yielded by a simple Bayes base caller.

The means for the read lengths were 200 and 400, the standard deviations were 10 % of the read lengths. For the pyrosequencing simulation, the parameters (see [4] for their meaning) were: Proportionality factor $k = 0.15$, error calculation was done with square root, background noise mean was 0.2, background noise standard deviation was 0.1.

S3 Future Work Details

S3.1 Incorporation Of Mate Pairs.

Mate pair information can simply be seen as a constraint on the resulting set of matches: Only matches satisfying the mate pair constraints should be considered. In extension, one could also consider all possible combination of matches for each read pair.

S3.2 Incorporation Of Quality Values.

Quality values could be incorporated by reordering matches as follows: For a sufficiently high value of k, all biologically interesting matches will be in the set of all feasible matches. Instead of using unit weight for mismatches and indels (as in the case of edit distance), each error is weighted. In the semi-global alignment of the read against the reference, each mismatching and inserted nucleotide is weighted by its quality value. Each deleted characters is weighted by the mean of its neighbouring bases.

Note, however, that this does not correspond to how Maq [3] and Bowtie handle qualities. Bowtie, for example, ignores any mismatches as long as the scores are below some threshold. Neither method handles qualities for indels.

Including quality values in the methodology is left as future work: There is no coherent handling of quality values in the literature and exploring different ways of including them would constitue work outside the scope of this manuscript.

S3.3 Incorporation of Smith-Waterman Scores.

Other scores like Smitch-Waterman (SW) scores can be incorporated in a similar way. Again, all biologically interesting matches under SW score will be contained in the set of all feasible edit distance matches for a sufficiently high value for k. We can compute the corresponding equivalence classes for these matches as proposed in this article and then re-weight them with the best SW score of all alignments in each class. Note that one would possibly have to chose an error rate larger than the 8 % used in this manuscript in order to guarantee to find all biologically relevant matches.

S4 Read Mapper Parametrization

This appendix describes the parametrization of the read mappers. Generally, we mapped the reads with a maximum of 8 % errors. The value was given as an error rate if supported, otherwise as an error count of $0.08 \times$ length of longest read, if possible.

Each read mapper was parametrized to output up to 100 alignments per read on real-world data and 1 alignment on simulated reads, if possible. In the evaluation, the best 100 alignments by edit distance were considered for each read for real-world data; ties were broken randomly. The limitation to one alignment is possible with all used read mappers, but not to arbitrary values with all read mappers.

Simulated paired-end reads were simulated with a normally distributed library size, $\mu = 1000$, $\sigma = 100$. All read mapper's library size error was set to $3 \cdot \sigma = 300$.

S4.1 Bowtie

Bowtie version 0.12.5 was used. First, the index is built:

```
bowtie-build GENOME.fasta GENOME.fasta
```

Default Parametrization: Bowtie Single-end mapping was performed without any special parameters. `--sam` makes Bowtie output SAM format, `-q` makes it read FASTQ files, `-k 100` allows up to 100 alignments per read.

```
bowtie --sam -q -k 100 GENOME.fasta READS.fastq OUTPUT.sam
```

For paired-end mapping on simulated data, the insert size was also given using `--minins` and `--maxins`:

```
bowtie --sam -q GENOME.fasta -1 LEFT.fastq -2 RIGHT.fastq \
  OUTPUT.sam --minins 700 --maxins 1300 -k 1
```

Parametrization With Improved Sensitivity: Bowtie* Following the suggestions by the authors of Bowtie, we tried various parameters to yield an optimized version, called *bowtie** in the evaluation. For short reads, changes from the default parameters lowered the result quality in terms of normalized intervals. For longer reads (> 50 bp), the following parametrization was performed:

We are setting the number of backtracking steps with `--maxbts 800`, the number of mismatches in the seeds with `--seedmms 3`, enable the *try hard mode* with `-y`, and set the sum of mismatching error qualities to 400 with `--maqerr`, such that at least 10 mismatches are ignored (40 is the cutoff for quality values). The additional parameters on the command line were:

```
--sam -q -k 100 --maxbts 800 --seedmms 3 -y --best --maqerr 400
```

We tried different values for these parameters. The change of the parameters from the ones mentioned above either increased the running time significantly but did not yield significantly better results or yielded significantly worse results (at a lower running time, of course), except for *-k* which we kept fixed to 100 so read mappers cannot benefit from creating arbitrarily many hits.

S4.2 Bwa

Bwa was used in version 0.5.8a. First, the index is built.

```
bwa index GENOME.fasta
```

454 reads were aligned as follows. The parameter `-n DISTANCE` sets the maximal allowed edit distance in alignments.

```
bwa bwasw -f OUTPUT.sam -n DISTANCE GENOME.fasta READS.fastq
```

For short reads, first SAI files have to be constructed, for paired-end reads one for the left and one for the right mates.

```
bwa aln -f TMP.sai -n DISTANCE GENOME.fasta READS.fastq
```

Then, single-end reads were processed as follows.

```
bwa samse -n 99 -f OUTPUT.sam -n DISTANCE GENOME.fasta TMP.sai  \
  READS.fastq
```

Simulated mate-pair reads were processed as follows.

```
bwa sampe -n 99 -f OUTPUT.sam -a 1300 -n DISTANCE  GENOME.fasta \
  LEFT.sai  RIGHT.sai LEFT.fastq RIGHT.fastq
```

The authors of Bwa suggested that default parameters should work well for all kinds data sets. We tried to adjust the parameters `-N`, `-o`, `-e`, `-l` and `-k` for `aln` but the result quality did only improve marginally, if at all, while running times increased, sometimes greatly.

Bwa prints only one line per read in the SAM file. Alternative alignments are stored in the extra `XA` field, which we expanded to additional lines for the evaluation.

S4.3 Shrimp2

Shrimp2 was run in version 2.1.1b. The general usage is as follows, the parameter `-E` enables SAM output, `-o 100` allows up to 100 matches per read. For Hamming, we are using the parameter `-U`.

The command line for single-end reads was:

```
gmapper -E -o 100 [-U] READS.fasta GENOME.fasta > OUTPUT.sam
```

The command line for paired-end, simulated reads was as follows. The parameter `-I 700,1300` sets the insert size, `-opp-in` sets the mate protocol, `-o 1` sets the number of alignments per read to 1.

```
gmapper -E -o 1 -p -p -opp-in -I 700,1300 READS.fasta \
  GENOME.fasta > OUTPUT.sam
```

The authors also suggested the non-standard setting `-H -s w16` which enables a weighted seed for longer reads to improve running time (in our tests, it also slightly improved normalized found intervals). For any sets of reads with reads of length > 50, we are also using this parameter. Furthermore, the authors suggested to tune the *window generation threshold*, but in our tests, this did not improve the result in terms of normalized found intervals.

We also tried to run Shrimp using the `--strata` option, however the program yielded worse results than with the parameters from above. Also, we tried to parametrize Shrimp to use edit distance (match score 0, mismatch score -1, gap open/extend score -1), but the program crashed with a floating point exception. Consequently, we ran it with the scores `--match 1 --mismatch -1000 --open-r -1000 --open-q -1000 --ext-r -1000 --ext-q -1000` but it also yielded worse scores than with default scores.

S4.4 Soap2

Soap2 was run in version 2.20. The index was built as follows:

```
2bwt-builder GENOME.fasta GENOME.fasta
```

Default Parametrization: Soap2 Single-end reads were mapped as follows. The parameter `-v` sets the number of mismatches that are allowed in one read.

```
soap -v DISTANCE -a READS.fastq -D GENOME.fasta.index -o TMP.soap
soap2sam_fastq.pl -a READS.fastq MP.soap > OUTPUT.sam
```

The Perl script *soap2sam_fastq.pl* is a modified version of the script *soap2-sam.pl* that Soap2 ships with. If the character N appears in the input Soap2 will replace it by one of the characters C, G, A, T. The script *soap2sam_fastq.pl* fixes this by taking the read sequence from the FASTQ input file.

```
soap -l 60 -r 2 -v DISTANCE -a LEFT.fastq -b RIGHT.fastq \
  -D GENOME.fasta.index -o MP.soap -2 UNPAIRED.soap      \
  -m 700 -x 1300
soap2sam_fastq.pl -p -a LEFT.fastq -b RIGHT.fastq        \
  MP.soap > OUTPUT.sam
```

Parametrization With Improved Sensitivity: Soap2* The parameter `-r 2` makes Soap2 output all repeat hits, even if it finds more than 100. The parameter `-v DISTANCE` allows up to DISTANCE mismatches in one read. DISTANCE is set to 8 % of the maximal read length. The parameter `-l 60` makes Soap2 first align the first 60 characters as a seed. This is recommended for long reads by the documentation and 60 appears to be a good value by our experiments. We tried out several other settings for `-r` and `-l` but there were only marginal improvements at substantially higher running times with other parametrizations.

Single-end reads were mapped as follows.

```
soap -l 60 -r 2 -v DISTANCE -a READS.fastq -D GENOME.fasta.index \
  -o TMP.soap
soap2sam_fastq.pl -a READS.fastq MP.soap > OUTPUT.sam
```

Paired-end reads were mapped as follows. `-m 700` and `-x 1300` set the insert size.

```
soap -l 60 -r 2 -v DISTANCE -a LEFT.fastq -b RIGHT.fastq \
  -D GENOME.fasta.index -o MP.soap -2 UNPAIRED.soap      \
  -m 700 -x 1300
soap2sam_fastq.pl -p -a LEFT.fastq -b RIGHT.fastq        \
  MP.soap > OUTPUT.sam
```

S5 Additional Figures

```
S      CG-AGCTTACTG
               X
R      CCTAGC-TCCTG
                X
read     TA-CTTCG
```

Figure S3: This figure shows the alignment of a read at its physical sampling position in R. Additionally, R is aligned to the reference sequence S.

```
reference   CCAGCG-AGAT    distance = 1
                  X
read           GCGC
```

Figure S4: This figure shows the alignment of a read at a gap in the reference sequence.

```
reference   CAACAACAACAACAA

reads         CAACAA
              CAACA-A
              CAACA--A
                 CAACAA
                 CAACA-A
                 CAACA--A
```

Figure S5: This figure shows an example of a read aligning against a repeat region in the reference sequence.

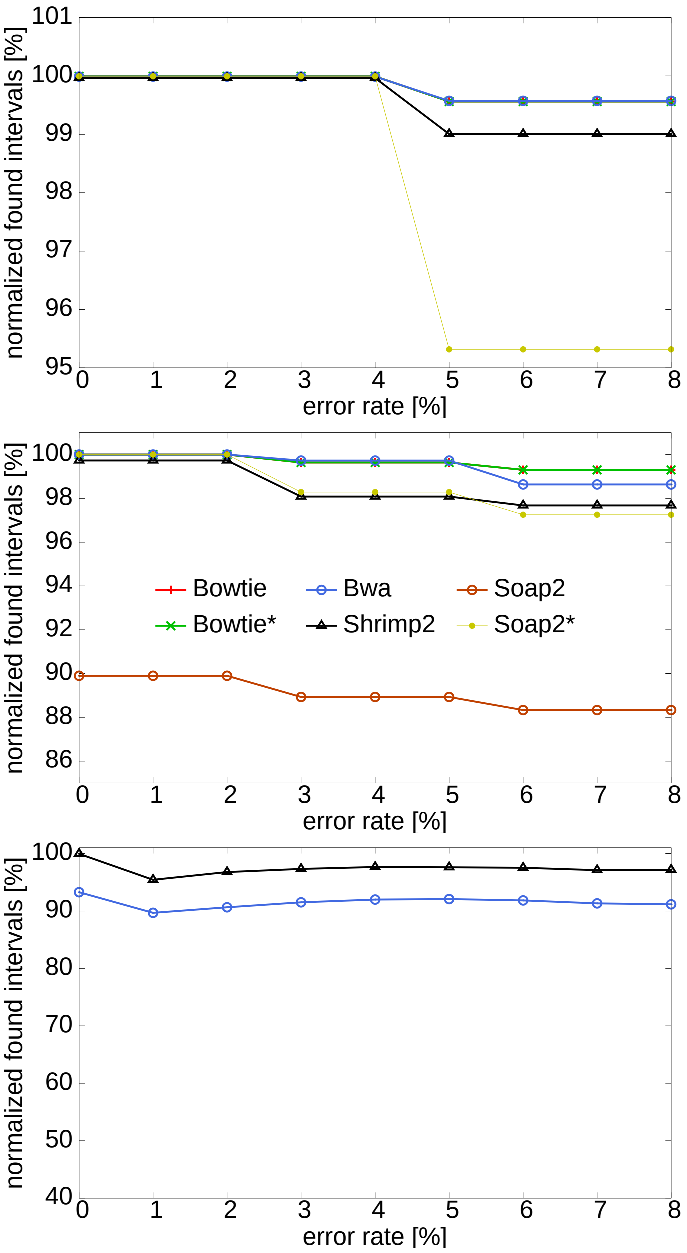

Figure S6: Normalized found intervals (percentage, category *all*) for different programs on reads ([EMBL:SRR000853], [EMBL:SRR003673], [EMBL:SRR038098]) from yeast. The gold standard was generated with an error rate of 8 % and edit distance. The x axis shows the variation of error rate in the evaluation. The key is only shown once and is the same for all plots. Note the different axis scalings.

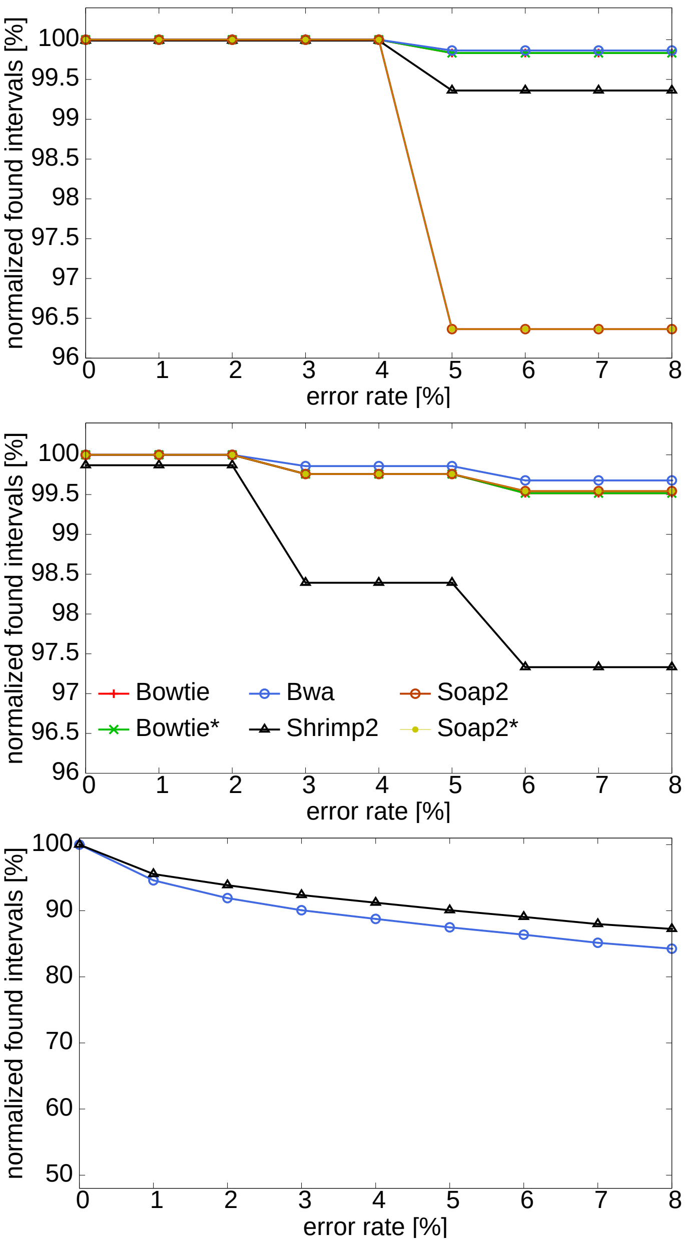

Figure S7: Normalized found intervals (percentage, category *any-best*) for different programs on reads ([EMBL:SRR000853], [EMBL:SRR003673], [EMBL:SRR038098]) from yeast. The gold standard was generated with an error rate of 8 % and edit distance. The x axis shows the variation of error rate in the evaluation. The key is only shown once and is the same for all plots. Note the different axis scalings.

S6 Additional Tables

	**Bowtie**	**Bwa**	**Soap2**	**Shrimp2**
algorithmic technique	FM-index based approximate string search	FM-index based approximate string search	FM-index based seed search and verification	q-gram counting and verification
distance measures supported	Hamming	Hamming, Edit, Smith-Waterman	Hamming with gaps allowed	Smith-Waterman
supported read length	≤ 1024	arbitrary	≤ 1024	arbitrary
supports quality value	Yes	Yes	No	No
supports full sensitivity mode	Not for suboptimal hits	Not for suboptimal hits	No, only for up to two mismatches	Adjustable to be fully sensitive, but many parameters need to be set

Table S1: This table shows some popular read mappers with some key properties. The example evaluation was performed with these tools. The full source code is freely available for all tools. Note that while the authors of Soap2 mention gap support in the publication, we could find no option to switch on gapped alignments. Furthermore, the provided script for conversion to the SAM format outputs ungapped alignments only.

sequencing technology	S. cerevisiae	D. melanogaster
454 LS	[EMBL:SRR000853] (267)	[EMBL:SRR034673] (273)
"short" Illumina	[EMBL:SRR038098] (20bp)	[EMBL:SRR026674] (36bp)
"long" Illumina	[EMBL:SRR003673] (36bp)	[EMBL:SRR049254] (100bp)

Table S2: For the combination of sequencing technology and species, this table gives the accession numbers of the reads used in the benchmark. The values in brackets are the lengths for Illumina reads and their means for 454 reads. The attributes "short" and "long" are relative to the length of the reads available in the SRA.

Genome	Size	Source
S. cerevisiae	12 Mbp	NC_001133-48, NC_001224
D. melanogaster	169 Mbp	flybase r5.29

Table S3: This table shows the genomes used in the benchmark and information about them.

	S. cerevisiae	D. melanogaster
Bowtie time [s]	17.0	454.9
memory [MB]	52.4	396.3
Bwa time [s]	8.6	200.8
memory [MB]	63.9	844.1
Soap2 time [s]	19.5	228.3
memory [MB]	273.6	440.4

Table S4: Running times and memory usage building the indices.

	[EMBL:SRR034673] 454, ⊘ 273 bp	[EMBL:SRR026674] Illumina, 36 bp	[EMBL:SRR049254] Illumina, 100 bp
Bowtie memory [MB]	–	144.7	145,8
time [s]	–	2.2	4.1
Bowtie* memory [MB]	–	144.7	191.9
time [s]	–	2.2	11.3
Bwa memory [MB]	246.4	130.0	235.9
time [s]	69.6	1.2	38.5
Shrimp2 memory [MB]	5,153.9	5,046.6	5,046.6
time [s]	848.9	368.0	349.0
Soap2 memory [MB]	–	951.2	953.2
time [s]	–	1.6	2.9
Soap2* memory [MB]	–	966.8	1,073.7
time [s]	–	2.7	11.0

Table S5: Running times and memory usage for read mappers on various read sets for D. melanogaster. Data is given for mapping in edit distance mode. Only Shrimp2 and Bwa were run on 454 reads. The times exclude any file conversion times. Missing entries in the memory rows are caused by too short running times hindering the memory usage measuring with `top`.

	[EMBL:SRR000853] 454, ⊘ 267 bp	[EMBL:SRR038098] Illumina, 20 bp	[EMBL:SRR003673] Illumina, 36 bp
Bowtie memory [MB]	–	20.0	20.0
time [s]	–	1.7	0.9
Bowtie* memory [MB]	–	20.0	–
time [s]	–	1.7	0.9
Bwa memory [MB]	29.4	–	–
time [s]	15.1	0.3	0.5
Shrimp2 memory [MB]	1,932.7	1,718.0	1,718.0
time [s]	44.2	29.8	32.9
Soap2 memory [MB]	–	–	–
time [s]	–	0.4	0.5
Soap2* memory [MB]	–	–	–
time [s]	–	0.5	0.5

Table S6: Running times and memory usage for read mappers on various read sets for S. cerivisiae. Data is given for mapping in edit distance mode. Missing entries in the memory rows are caused by too short running times hindering the memory usage measuring with `top`. The times exclude any file conversion times.

	Illumina 36 bp	Illumina 50 bp	Illumina 100 bp	454 ⊘ 200 bp	454 ⊘ 400 bp
Bowtie	82.3	75.0	57.6	–	–
Bowtie*	82.3	75.0	61.6	–	–
Bwa	96.4	95.9	96.2	–	–
Shrimp2	94.9	94.7	95.7	92.2	93.9
Soap2	82.4	74.8	57.1	–	–
Soap2*	82.4	74.8	59.6	–	–

Table S7: Performance in found interval percentage for the read mappers on simulated S. cerivisiae reads. Data is given for mapping in edit distance mode. Only Shrimp2 was run on simulated 454 mate-paired reads.

References

[1] M. Holtgrewe. Mason – a read simulator for second generation sequencing data. Technical Report TR-B-10-06, Institut für Mathematik und Informatik, Freie Universität Berlin, October 2010.

[2] Jan O Korbel, Alexej Abyzov, Xinmeng Jasmine Mu, Nicholas Carriero, Philip Cayting, Zhengdong Zhang, Michael Snyder, and Mark B Gerstein. Pemer: a computational framework with simulation-based error models for inferring genomic structural variants from massive paired-end sequencing data. *Genome Biol*, 10(2):R23, 2009.

[3] Heng Li, Jue Ruan, and Richard Durbin. Mapping short DNA sequencing reads and calling variants using mapping quality scores. *Genome Res.*, 18(11):1851–1858, 2008.

[4] Daniel C Richter, Felix Ott, Alexander F Auch, Ramona Schmid, and Daniel H Huson. Metasim: a sequencing simulator for genomics and metagenomics. *PLoS One*, 3(10):e3373, 2008.
